# LncRNA NORAD promotes bone marrow stem cell differentiation and proliferation by targeting miR-26a-5p in steroid-induced osteonecrosis of the femoral head

**DOI:** 10.1186/s13287-020-02075-x

**Published:** 2021-01-07

**Authors:** Dapeng Fu, Sheng Yang, Jianmin Lu, Haoyi Lian, Kairong Qin

**Affiliations:** 1 Department of Orthopaedics, Affiliated Zhongshan Hospital of Dalian University, Dalian, 116001 Liaoning People’s Republic of China; 2 Department of Biomedical Engineering, Dalian University of Technology, Dalian, 116024 Liaoning People’s Republic of China

**Keywords:** Steroid-induced osteonecrosis of the femoral head, LncRNA NORAD, miR-26a-5p, OPG/RANK/ RANKL pathway, Proliferation

## Abstract

**Background:**

Steroid-induced osteonecrosis of the femoral head (SONFH) is a devastating orthopedic disease, which seriously affects the quality of life of patients. The study aims to investigate the effects of LncRNA NORAD on SONFH.

**Methods:**

Human bone marrow-derived mesenchymal stem cells (hBMSCs) were isolated from the proximal femur of patients during routine orthopedic surgery and then cultured with dexamethasone (Dex) and transfected with NORAD overexpression vector, siRNA-NORAD and miR-26a-5p mimics. The mRNA expression of NORAD, miR-26a-5p, OPG, RANK, and RANKL was detected by RT-qPCR. Cell proliferation and apoptosis was measured by CCK-8 assay and flow cytometry, respectively. The protein expression of RUNX2, OPG, RANK, and RANKL was detected by western blot. The dual-luciferase reporter gene assay was performed to confirm the binding between NORAD and miR-26a-5p.

**Results:**

NORAD expression was downregulated in SONFH tissues, while miR-26a-5p expression was upregulated. Overexpression of NORAD improved DEX-induced inhibition of proliferation and differentiation, and promotion of apoptosis in hBMSCs, while knockdown of NORAD led to the opposite results. Moreover, NORAD improved DEX-induced inhibition of proliferation and differentiation, and promotion of apoptosis by regulation of miR-26a-5p in hBMSCs.

**Conclusions:**

NORAD expression was downregulated in SONFH tissues, while miR-26a-5p expression was upregulated. NORAD improved DEX-induced inhibition of proliferation and differentiation, and promotion of apoptosis by regulation of miR-26a-5p in hBMSCs.

## Background

Osteonecrosis of the femoral head (ONFH) is characterized by the collapse of the femoral head due to bone cells and bone marrow to be necrotic [1, 2]. At present, glucocorticoid (GC) has been commonly used to treat rheumatic, autoimmune, inflammation-dependent diseases and hematopoietic system diseases [3]. However, the usage of GC is one of the most common causes of ONFH [4]. Clinical observation confirmed that most patients who were treated with high-dose GC appeared clinical symptoms of osteonecrosis within 2 years [1]. Moreover, glucocorticoid-induced osteonecrosis of the femoral head (GIOFH) usually involves young adults and most patients with GIOFH require surgery, which seriously affects the quality of life of patients [5, 6]. However, the pathogenesis of steroid-induced osteonecrosis of the femoral head (SONFH) is unclear.

GC can inhibit the bone formation by affecting bone cell [3]. One study has been indicated that the change of osteogenic differentiation ability of mesenchymal stem cells (MSCs) is the cause of the imbalance of necrosis and bone regeneration, which is a key factor in the pathogenesis of non-traumatic ONFH [7]. Bone marrow mesenchymal stem cells (BMSCs) are a group of pluripotent stem cells with multidirectional differentiation potential capabilities [8, 9]. Osteoblast, chondroblast, and adipocyte can be derived from BMSCs, which may further secrete growth factors to promote tissue regeneration [10]. Human bone marrow-derived mesenchymal stem cells (hBMSCs) are characterized by a negative phenotype of hematopoietic lineage, which are derived from the human bone marrow cavity [1]. Recent studies have shown that the occurrence and development of many orthopedic diseases are closely related to hBMSCs [7, 11]. Therefore, the analysis of the potential mechanisms of aberrant osteogenic differentiation of BMSCs may play an important role in the treatment of SONFH.

Long noncoding RNAs (lncRNAs) are RNAs that lack the ability of protein coding and their length are more than 200 nucleotides [12]. In recent years, numerous studies have demonstrated that lncRNAs play a very important role in cell proliferation, osteogenic differentiation, apoptosis, and autophagy [13–15]. LncRNA NORAD, as a long noncoding RNA activated by DNA damage, expresses abnormally in many kinds of tumors and serves as an oncogene [16–18]. Xu et al. [19] have reported that NORAD promote the proliferation, invasion, migration, and EMT of ovarian cancer cells. Wang et al. [20] have found that knockdown of NORAD suppressed osteosarcoma cell proliferation and in vivo explant growth. In addition, Tao et al. [21] have revealed that NOARD can inhibit the apoptosis of gastric cancer cells by down-regulating caspase-3. Additionally, it also has been reported that NORAD attenuates endothelial cell apoptosis [22]. Micro RNAs (miRNAs), as non-coding single-stranded RNA, are involved in proliferation, apoptosis, differentiation, and so on [23–25]. Studies have revealed that the interaction of lncRNAs and miRNAs can affect the osteogenic differentiation of BMSCs [26]. Li et al. [27] have found that upregulation of miR-26a-5p inhibits osteogenesis and downregulation of miR-26a-5p promotes osteogenesis. However, the relationship between NORAD and miR-26a-5p in SONFH is not reported.

In this study, we detected the expression of NORAD and miR-26a-5p in SONFH tissues. In addition, we explored the biological effects of NORAD and miR-26a-5p on cell proliferation, apoptosis, and OPG/RANK/RANKL pathway in hBMSCs. Our findings may provide a new target direction for the treatment and diagnosis of SONFH.

## Material and methods

### Subjects and specimen collection

Bone marrow samples were obtained from 20 patients with SONFH as well as 20 patients with femoral neck fracture who underwent surgeries between April 2018 and March 2020 at our hospital. According to the Steinberg or University of Pennsylvania system, diagnosis of SONFH was proved by preoperative radiographs and magnetic resonance image (MRI). All patients with SONFH who had a history of taking a greater than 1800 mg of GCs or a long-term glucocorticoid therapy for more than 4 weeks were included, and patients concurrent with cardiovascular diseases, congenital diseases, or tumor-related diseases were excluded in the present study. In addition, patients with femoral neck fracture have no history of GC therapy. All participants were signed written informed consent, and our study was approved by the ethics committee of our hospital.

### Isolation and culture of hBMSCs

Bone marrow aspirates (10 ml) were obtained from the proximal femur of patients during routine orthopedic surgery. The aspirates were resuspended in phosphate-buffered saline (PBS), and then, the cell suspension was injected into a centrifuge tube containing an equal volume of lymphocyte separation solution. After centrifugation for 30 min at 2000 r/min, the mononuclear cells in the white layer were collected and cultured in low-glucose Dulbecco’s modified Eagle’s medium (DMEM-LG, Gibco, Rockville, MD, USA) with 10% fetal bovine serum (FBS, Gibco) and Penicillin-Streptomycin solution (Invitrogen, Carlsbad, CA, USA) at 37 °C in 5% CO_2_. For inhibition of differentiation, the cells were treated with different concentration of dexamethasone (DEX;10^−8^, 10^−7^, and 10^−6^ M; Sigma).

### Cell transfection

BMSCs were divided into control (without treatment), Dex (BMSCs were treated with 10^−6^ Dex), Dex+Vector [BMSCs were treated with 10^-6^ Dex and transfected with negative control of NORAD (Vector)], Dex+NORAD (BMSCs were treated with 10^−6^ Dex and transfected with NORAD overexpression vector), Dex+si-NC [BMSCs were treated with 10^−6^ Dex and transfected with negative control of NORAD (si-NC)], Dex+si1-NORAD (BMSCs were treated with 10^−6^ Dex and transfected with siRNA1-NORAD), Dex+Vector+mimics NC (BMSCs were treated with 10^−6^ Dex and transfected with Vector and mimics NC), and Dex+NORAD+miR-26a-5p mimics (BMSCs were treated with 10^−6^ Dex and transfected with NORAD overexpression vector and miR-26a-5p mimics) group. NORAD, si-NORAD, miR-26a-5p mimics, and corresponding negative control were synthesized by GenePharma (Shanghai, China). The transfection was performed by Lipofectamine 2000 (Invitrogen) according to the manufacturer’s instructions. Reverse transcription-quantitative polymerase chain reaction (RT-qPCR) was used to determine the transfection efficiency.

### RT-qPCR assay

According to manufacturer’s protocol, TRIzol reagent (Invitrogen) was used to extract total RNA from tissues and cells. Total RNA was taken for reverse transcription of cDNA by a PrimeScript RT-PCR Kit (Takara, Dalian, China). Subsequently, RT-PCR was performed by the SYBR Green PCR kit (Applied Biosystems, Shanghai, China) on ABI 7500 real-time PCR system (Applied Biosystems). The relative mRNA expression was calculated by 2^−ΔΔCt^ method, and β-actin or U6 was used as the internal reference. Primer sequences (Table 1) were synthesized by Invitrogen.

**Table 1:** The sequences of primers

Primers	Sequences(5′-3′)
NORAD-F	TGATAGGATACATCTTGGACATGGA
NORAD-R	AACCTAATGAACAAGTCCTGACATACA
OPG-F	TTGCACCACTCCAAATCCAG
OPG-R	AATCGCACCCACAACCG
RANK-F	GTCTCATCGTCCTGCTCCTCTT
RANK-R	CAGCGTTTTCCCTCCCTTC
RANKL-F	GCAGCATCGCTCTGTTCCTGTA
RANKL-R	GCATGAGTCAGGTAGTGCTTCTGTG
β-actin-F	AGACCACCTTCAACTCGATCAT
β-actin-R	ACTCGTCATACTCCTGCTTGCT
miR-26a-5p-F	CGCGAATTCTTGAGGTGAGGCTCAGGAGG
miR-26a-5p-R	ACGGGATCCTTGGCTACAGGCAAAGGGTT
U6-F	CTCGCTTCGGCAGCAGCACATATA
U6-R	AAATATGGAACGCTTCACGA

### Western blot analysis

Radioimmunoprecipitation assay (RIPA) buffer (Sigma-Aldrich, St. Louis, MO, USA) was used to extract total protein from BMSCs. BCA assay (Beyotime, Nanjing, China) was used to detect the total protein concentration. Equal quantities of protein (30 μg) were subjected to 10% sodium dodecyl sulphate-polyacrylamide gel electrophoresis and transferred onto polyvinylidene fluoride membranes (Millipore, Billerica, MA,USA). After being blocked with 5% fat-free milk, the membranes were incubated with primary antibody (RUNX2, ab192256; OPG, ab73400; RANK, ab200369; RANKL, ab9957; GAPDH, ab181602. 1: 1000. Abcam, Cambridge, MA, USA) at 4 °C overnight. Next, the membranes were incubated with the HRP labeled second antibody (1:5000; Wuhan Boster Biological Technology, ltd., Wuhan, china) at 37 °C for 1 h. The ECL system (Pierce, Rockford, IL, USA) was used to visualize the protein bands, and image lab version 3.0 (Bio-Rad Laboratories, Inc.) was used to analyze the intensity of the bands.

### Dual-luciferase reporter gene assay

The potential binding site between NORAD and miR-26a-5p was predicted by StarBase (http://starbase.sysu.edu.cn/index.php), and relevant primers (NORAD-MT and NORAD-WT) were synthesized by Shanghai GenePharma. HEK293 cells were seeded in 96-well plates until grew into about 85% concentration, miR-26a-5p mimics, mimics NC, NORAD-MT, or NORAD-WT were co-transfected into cells by the Lipofectamine 2000 reagent. After transfection for 48 h, Dual-Luciferase Reporter Gene Assay Kit (Beyotime) was used to detect the relative fluorescence intensity.

### Cell proliferation assay

The proliferation ability of hBMSCs was measured by the Cell Counting Kit-8 (KeyGEN Biotech Inc., Shanghai, China) assay. Briefly, BMSCs (3 × 104 cell/ml) were seeded in 96-well plates after corresponding treatment. After incubation for 0, 24, 48, and 72 h, cells were incubated with 10 μl CCK-8 solution for 2 h at 37 °C and 5% CO_2_. Subsequently, the optical density (OD) value at 490 nm was measured by a spectrophotometric microplate reader (Bio-Rad, Hercules, CA,USA).

### Cell apoptosis assay

Cell apoptosis was estimated by Annexin V-FITC/PI Apoptosis Assay Kit (Sangon Biotech, Shanghai, China). After corresponding treatment, BMSCs were incubated with 10 μl Annexin V-FITC and 5 μl propidium iodide (PI) for 30 min in the dark. Then, the flow cytometry (BD Biosciences, Franklin Lakes, NJ, USA) was used to analyze cell apoptosis.

### Statistical analysis

All experimental data were expressed as mean ± standard deviation (SD), and GraphPad Prism 7.0 software (USA) was used to perform statistical analysis. One-way ANOVA was used to analyze the statistical differences among three or more groups. Unpaired Student’s *t* test was used to analyze the statistical differences between two groups. *P* value less than 0.05 was considered as statistically significant.

## Results

### LncRNA NORAD expression is downregulated in SONFH tissues and Dex-treated hBMSCs, and treatment of Dex inhibits proliferation and the OPG/RANK/RANKL pathway in hBMSCs

**Fig. 1:**
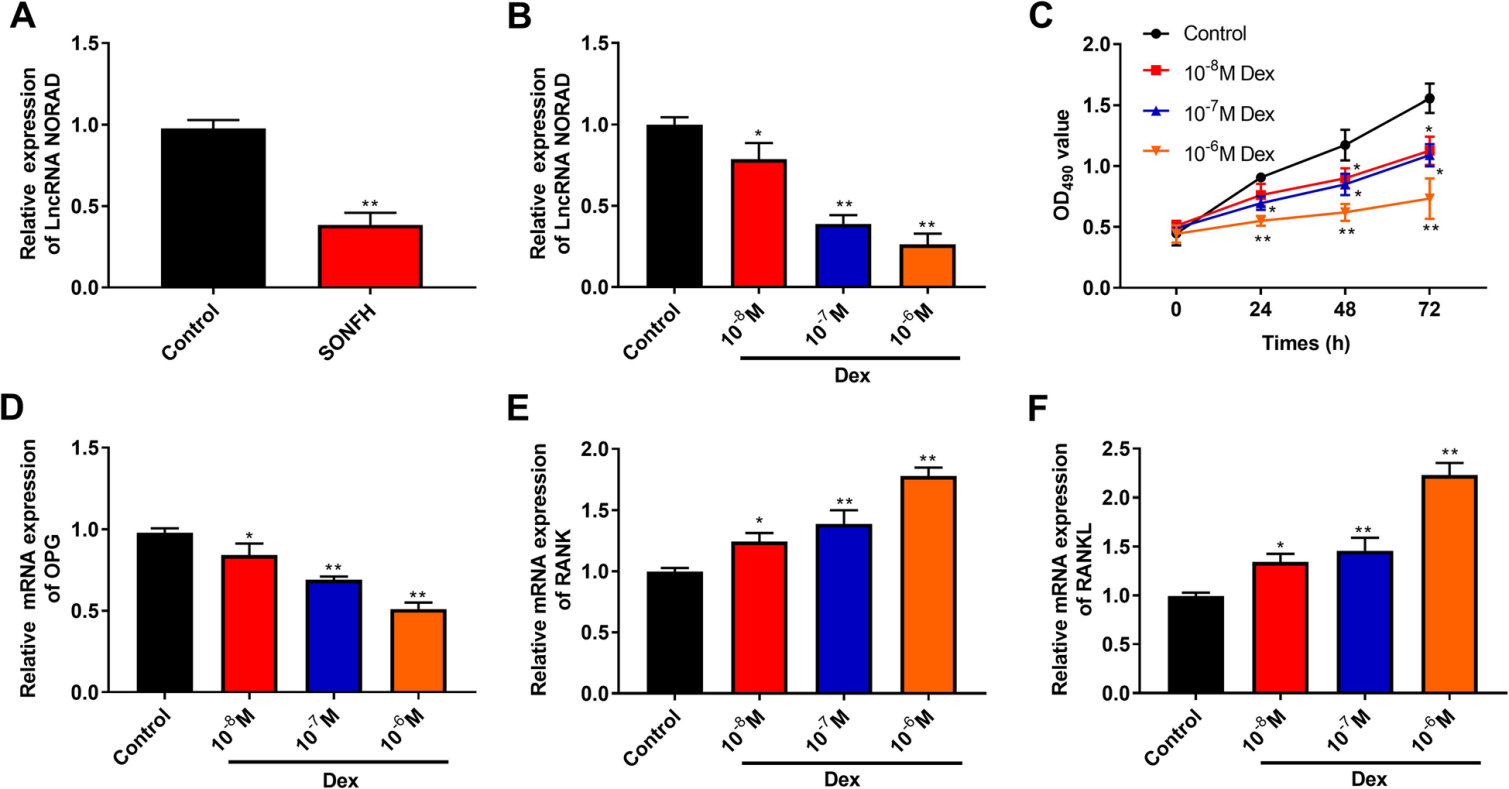
LncRNA NORAD expression was downregulated in SONFH tissues and Dex-treated hBMSCs, and treatment of Dex inhibited proliferation and the OPG/RANK/RANKL pathway in hBMSCs. **a** The NORAD expression in bone marrow samples from patients with SONFH (*n* = 20) and femoral neck fracture (control, *n* = 20) was detected by RT-qPCR. **b** The NORAD expression in different concentration of Dex-treated hBMSCs was detected by RT-qPCR. **c** The proliferation ability of hBMSCs was measured by the CCK-8 assay. **d**–**f** The mRNA expression of OPG (**d**), RANK (**e**), and RANKL (**f**) was detected by RT-qPCR. *P* < 0.01 and *P* < 0.05 compared with the control group

We investigated the NORAD expression in bone marrow samples from patients with SONFH and femoral neck fracture (control) by RT-qPCR. As shown in Fig. 1a, NORAD expression in SONFH was lower than that in control group (*P* < 0.01). Next, we investigated the effects of Dex (0, 10^−6^, 10^−7^, and 10^−8^ M) on NORAD expression, proliferation, and OPG/RANK/RANKL pathway in hBMSCs. As presented in Fig. 1b, Dex reduced NORAD expression in a dose-dependent manner. In addition, Dex inhibited hBMSC proliferation (OD_490_ value) by increase in concentration and the time of exposure (Fig. 1c, *P* < 0.01). The mRNA expression of OPG, RANK, and RANKL was also detected by RT-qPCR. The results showed that the mRNA expression of OPG was decreased, and the mRNA expression of RANK and RANKL was increased in a dose-dependent manner (*P* < 0.05, Fig. 1d–f).

### Overexpression of NORAD improves Dex-induced inhibition of proliferation and differentiation, and promotion of apoptosis in hBMSCs

**Fig. 2:**
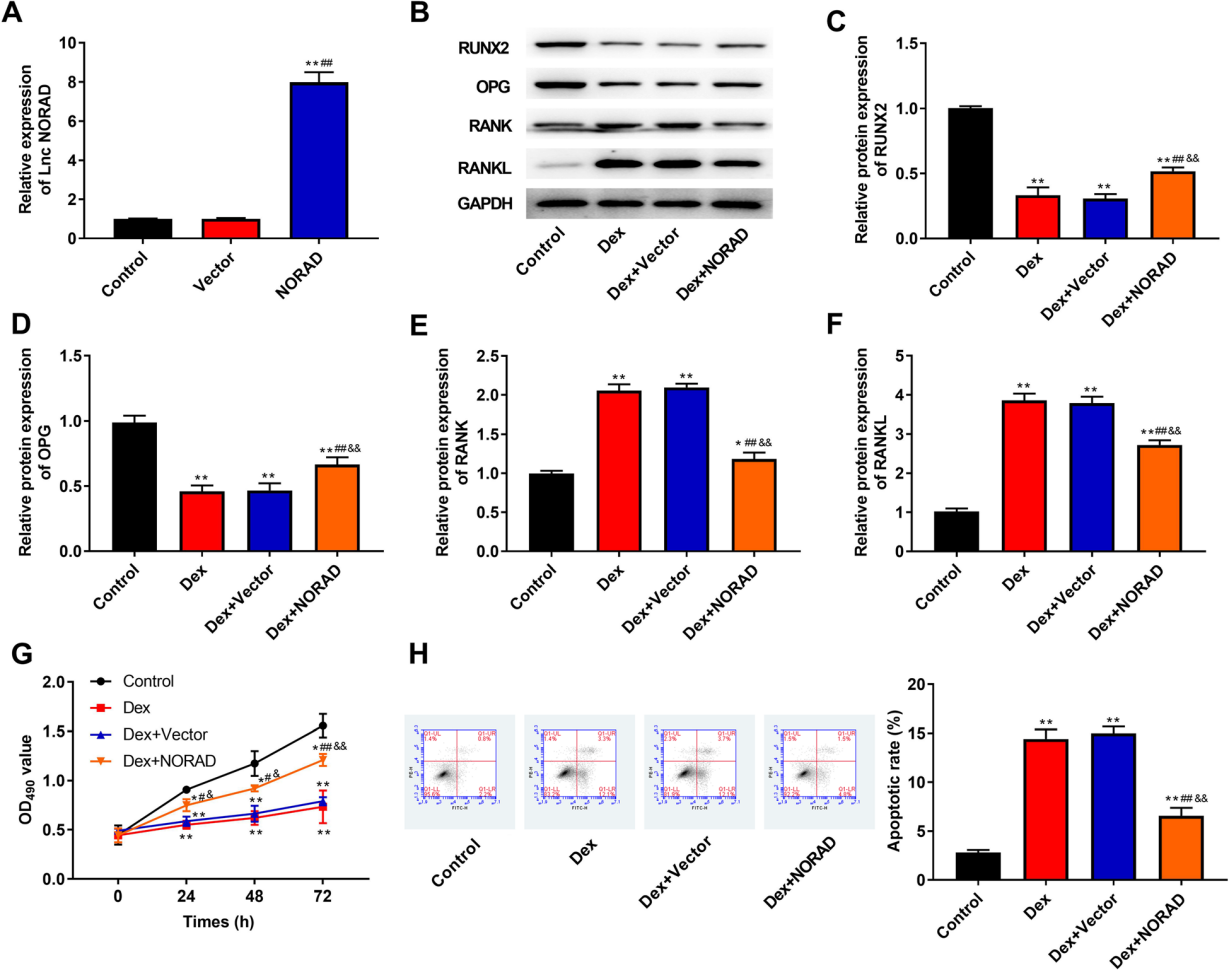
Overexpression of NORAD improved Dex-induced inhibition of proliferation and differentiation, and promotion of apoptosis in hBMSCs. **a** The NORAD expression in hBMSCs was detected by RT-qPCR. **b** The bands of RUNX2, OPG, RANK, and RANKL protein was detected by western blot. **c**–**f** The protein expression of RUNX2 (**c**), OPG (**d**), RANK (**e**), and RANKL (**f**) was detected by western blot. **g** The proliferation ability of hBMSCs was measured by the CCK-8 assay. **h** The flow cytometry was used to detect apoptosis in hBMSCs. **P < 0.01 and *P < 0.05 compared with control group, ^##^*P* < 0.01 and ^#^*P* < 0.05 compared with Dex and Vector group, ^&&^*P* < 0.01 and ^&^*P* < 0.05 compared with Dex+Vector group

To investigate the role of NORAD in Dex-induced hBMSCs’ osteoblastic differentiation, proliferation, and apoptosis, NORAD was overexpressed in hBMSCs. The results of RT-qPCR showed that NORAD expression was significantly increased when cells were transfected with NORAD overexpression vector, which suggested the transfection was successful (*P* < 0.01, Fig. 2a). As shown in Fig. 2b–f, the protein expression of RUNX2 and OPG which was decreased by Dex was increased after NORAD overexpression (*P* < 0.01), while the protein expression of RANK and RANKL which was increased by Dex was reduced after NORAD overexpression (*P* < 0.01). Moreover, when NORAD was overexpressed, Dex-induced proliferation reduction was attenuated (*P* < 0.05, Fig. 2g). As can be seen in Fig. 2h, Dex increased apoptotic rate compared with control group (*P* < 0.01), but Dex-induced apoptosis increase was attenuated by overexpression of NORAD (*P* < 0.01).

### Knockdown of NORAD aggravates Dex-induced inhibition of proliferation and differentiation, and promotion of apoptosis in hBMSCs

**Fig. 3:**
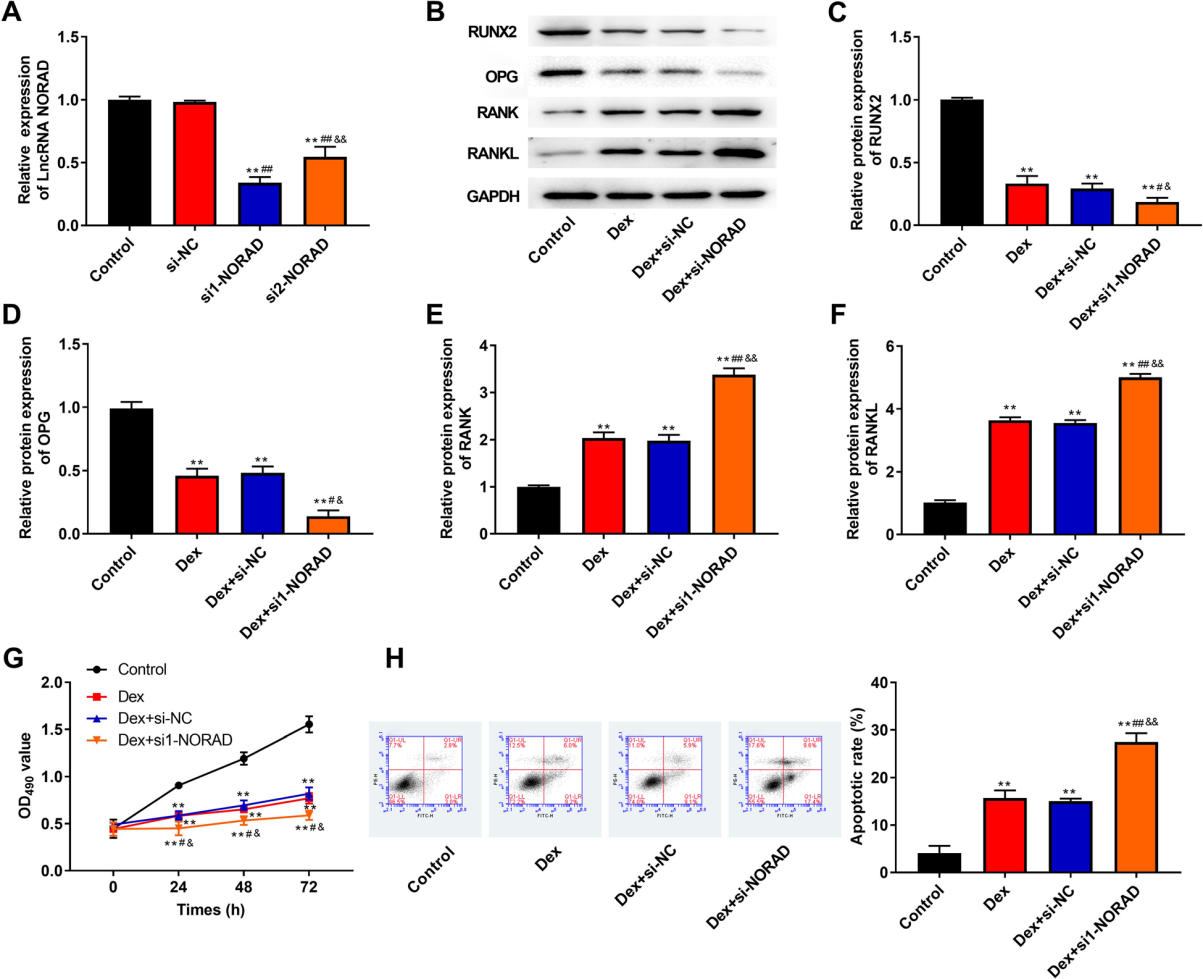
Knockdown of NORAD aggravated Dex-induced inhibition of proliferation and differentiation, and promotion of apoptosis in hBMSCs. **a** The NORAD expression in hBMSCs was detected by RT-qPCR. **b** The bands of RUNX2, OPG, RANK, and RANKL protein was detected by western blot. **c**–**f** The protein expression of RUNX2 (**c**), OPG (**d**), RANK (**e**), and RANKL (**f**) was detected by western blot. **g** The proliferation ability of hBMSCs was measured by the CCK-8 assay. **h** The flow cytometry was used to detect apoptosis in hBMSCs. **P < 0.01 compared with control group, ^##^*P* < 0.01 and ^#^*P* < 0.05 compared with Dex and si-NC group, ^&&^*P* < 0.01 and ^&^*P* < 0.05 compared with Dex+si-NC group

We hypothesized that knockdown of NORAD aggravated Dex-induced inhibition of proliferation and promotion of apoptosis in hBMSCs. To confirm this hypothesis, NORAD was low expressed by transfection with siRNA-NORAD in hBMSCs. As shown in Fig. 3a, NORAD expression in siRNA1-NORAD and siRNA2-NORAD group was significantly decreased compared with Control and si-NC group (*P* < 0.01), which suggested the transfection was successful. Additionally, knockdown of NORAD significantly decreased the protein expression of RUNX2 and OPG decreased by Dex (*P* < 0.05, Fig. 3b–d), but knockdown of NORAD significantly increased the protein expression of RANK and RANKL increased by Dex (*P* < 0.01, Fig. 3b, e, f). By knockdown of NORAD, proliferation was reduced compared with Dex and Dex + si-NC group (*P* < 0.05, Fig. 3g). In addition, Dex-induced apoptotic rate increase was aggravated by siRNA1-NORAD transfection in hBMSCs (*P* < 0.01, Fig. 3h).

### miR-26a-5p is a target and is negatively regulated by NORAD

**Fig. 4:**
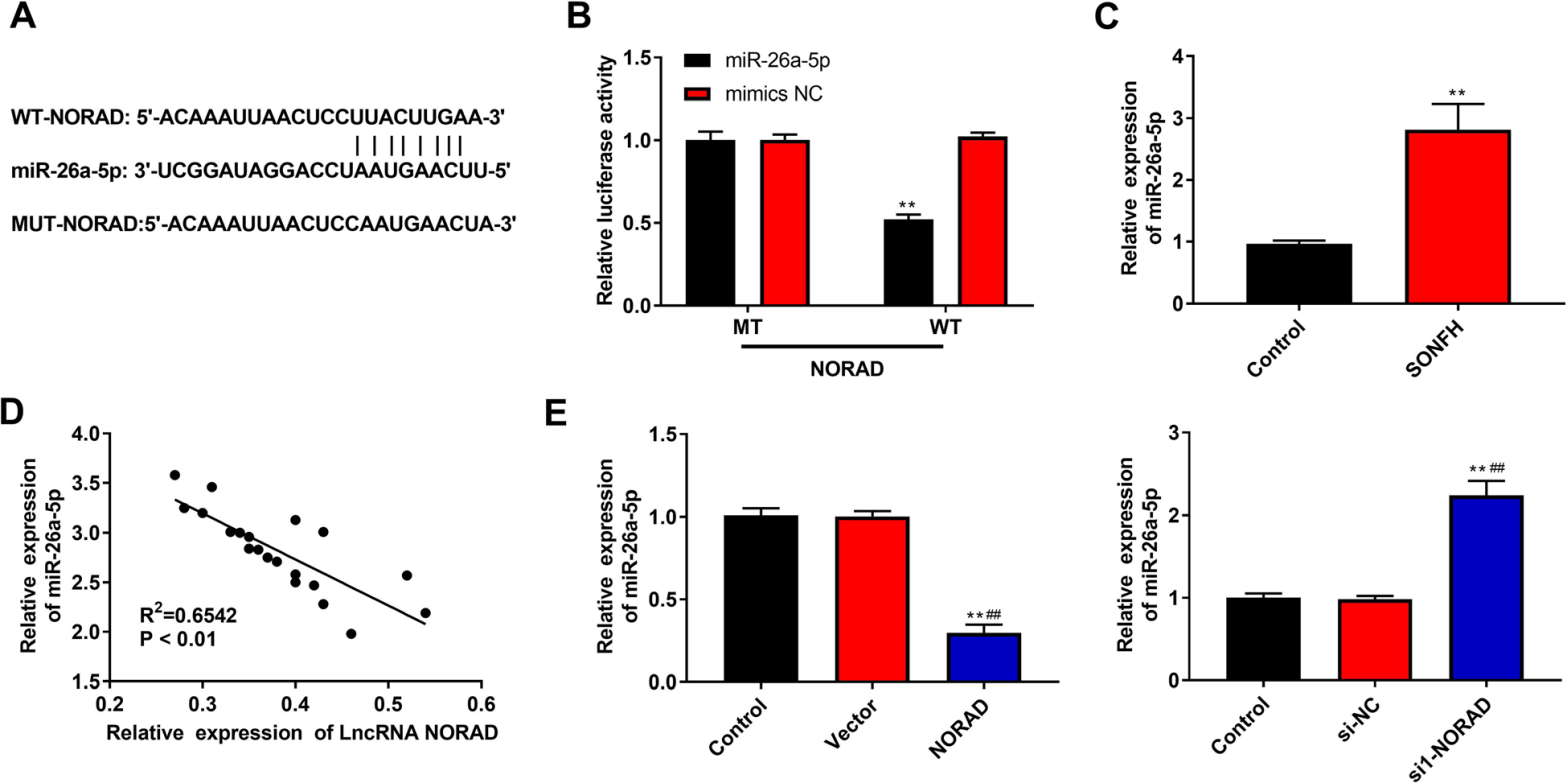
miR-26a-5p was a target and was negatively regulated by NORAD. **a** StarBase online provided the putative binding sites of NORAD and miR-26a-5p. **b** Dual-luciferase reporter gene assay was performed to confirm the binding between NORAD and miR-26a-5p. **c** The miR-26a-5p expression in bone marrow samples from patients with SONFH (*n* = 20) and femoral neck fracture (control, *n* = 20) was detected by RT-qPCR. **d** The correlation between NORAD expression and miR-26a-5p expression in patients with SONFH was analyzed by Spearman’s correlation analysis. **e** The miR-26a-5p expression in hBMSCs was detected by RT-qPCR. **P < 0.01 compared with control group, ^##^*P* < 0.01 compared with Vector and si-NC group

In order to explore the mechanisms of NORAD-mediated progression in SONFH, we used StarBase to predict the downstream targets of NORAD. The results showed that NORAD might have direct effect on miR-26a-5p (Fig. 4a). Next, dual-luciferase reporter gene assay was performed to further confirm the prediction. As shown in Fig. 4b, when HEK293 cells were co-transfected with miR-26a-5p mimics and NORAD-WT, the luciferase activity was remarkably reduced compared with co-transfection of NORAD-WT and miR-145 mimics NC, while there were no significant difference in NORAD-MT, which was indicated that miR-26a-5p was a target of NORAD. In addition, miR-26a-5p expression in SONFH was higher than that in Control group (*P* < 0.01, Fig. 4c), and NORAD and miR-26a-5p expression represented a negative correlation (*P* < 0.01, Fig. 4d). Moreover, it was found that miR-26a-5p was downregulated in hBMSCs transfected with NORAD overexpression vector (*P* < 0.01, Fig. 4e), while knockdown of NORAD led to the opposite results.

### NORAD improves Dex-induced inhibition of proliferation and differentiation, and promotion of apoptosis by regulation of miR-26a-5p in hBMSCs

**Fig. 5:**
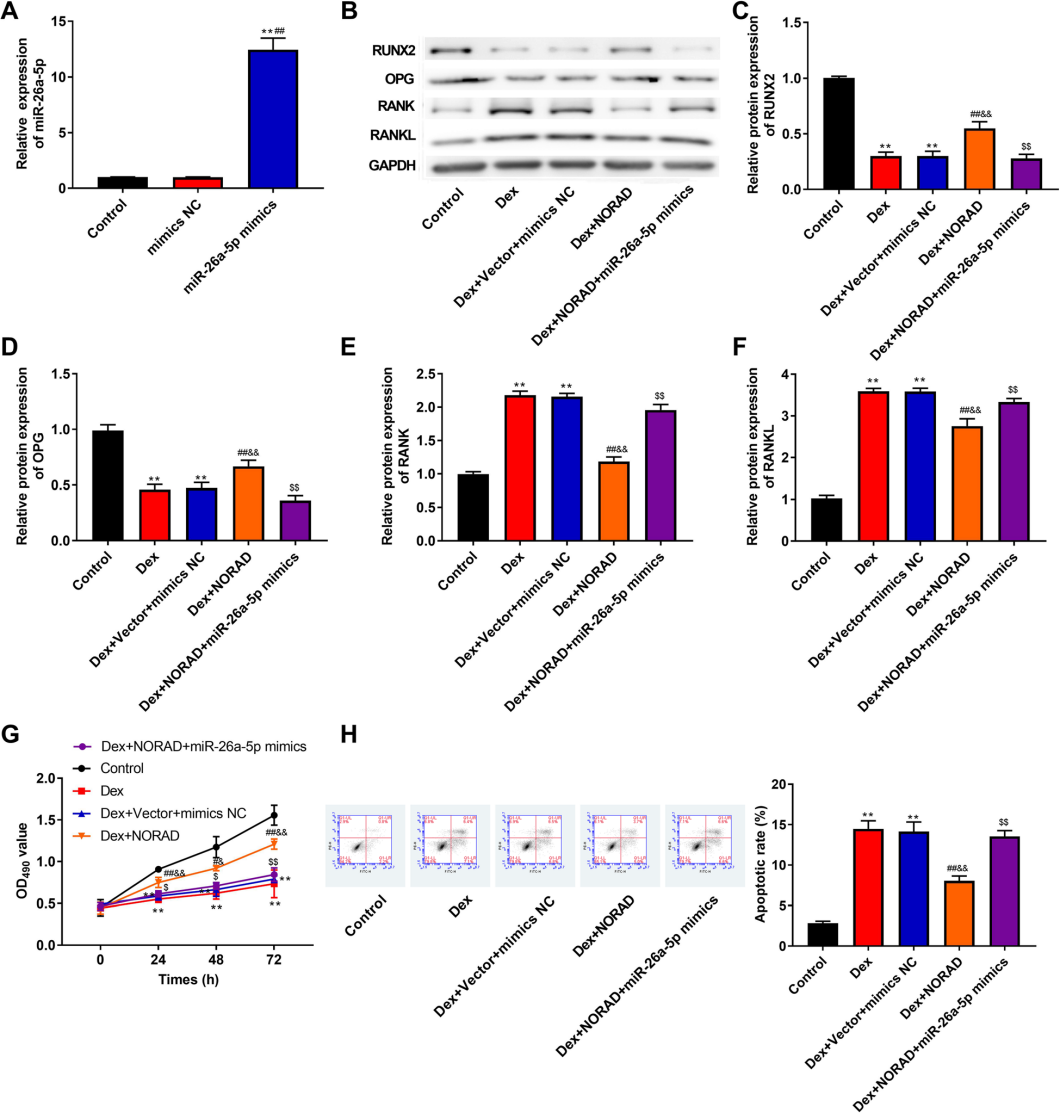
NORAD improved Dex-induced inhibition of proliferation and differentiation, and promotion of apoptosis by regulation of miR-26a-5p in hBMSCs. **a** The miR-26a-5p expression in hBMSCs was detected by RT-qPCR. **b** The bands of RUNX2, OPG, RANK, and RANKL protein was detected by western blot. **c**–**f** The protein expression of RUNX2 (**c**), OPG (**d**), RANK (**e**), and RANKL (**f**) was detected by western blot. **g** The proliferation ability of hBMSCs was measured by the CCK-8 assay. **h** The flow cytometry was used to detect apoptosis in hBMSCs. **P < 0.01 compared with control group, ^##^*P* < 0.01 and ^#^*P* < 0.05 compared with Dex group, ^&&^*P* < 0.01 and ^&^*P* < 0.05 compared with Dex+Vector+ mimics NC group, ^$$^*P* < 0.01 and ^$^*P* < 0.05 compared with Dex+NORAD group

To investigate whether NORAD exerted its effects by regulating miR-26a-5p, NORAD overexpression vector and miR-26a-5p mimics were co-transfected into hBMSCs. As shown in Fig. 5a, miR-26a-5p expression was increased when cells were transfected with miR-26a-5p mimics, which suggested the transfection was successful (*P* < 0.01). As illustrated in Fig. 5b–f, the protein expression of RUNX2 and OPG was increased and the protein expression of RANK and RANKL was decreased by overexpression of NORAD (*P* < 0.01), while transfection of miR-26a-5p mimics remarkably inhibited these effects (*P* < 0.01). In addition, as can be seen in Fig. 5g, h, proliferation ability was greatly enhanced but cell apoptosis was repressed by overexpression of NORAD (*P* < 0.05), while these effects were weakened by overexpression of miR-26a-5p (*P* < 0.05). Taken together, the above findings suggested that NORAD improved Dex-induced inhibition of proliferation and differentiation, and promotion of apoptosis by regulation of miR-26a-5p in hBMSCs.

## Discussion

Bone cells’ death caused by blood circulation disorders lead to the occurrence of ONFH [28]. ONFH is divided into traumatic and non-traumatic, and the use of long-term high-dose GC caused most non-traumatic necrosis [1]. Recently, many studies have shown that BMSCs play an important role in the development of ONFH [29, 30]. Meanwhile, more and more evidences have showed that various lncRNAs and miRNAs have regulatory effects in the progress of the ONFH [31–33]. NORAD has been confirmed to be overexpressed in various cancers, and it is involved in proliferation, differentiation, and apoptosis [16, 34, 35]. In the present study, we found that NORAD expression was downregulated in SONFH tissues, while miR-26a-5p expression was upregulated. Overexpression of NORAD improved Dex-induced inhibition of proliferation and differentiation, and promotion of apoptosis in hBMSCs, while knockdown of NORAD led to the opposite results. Moreover, NORAD improved Dex-induced inhibition of proliferation and differentiation, and promotion of apoptosis by regulation of miR-26a-5p in hBMSCs.

The dysregulation of osteogenesis differentiation is an important pathway in SONFH; thus, BMSCs have become a research hotspot and are considered to be potential in treatment of SONFH [36]. Although some development of SONFH have made in clinical and basic researches, its molecular mechanisms are still unclear. In recent years, there are more and more report about the role of lncRNAs in osteogenesis. For example, Wei et al. [37] have demonstrated that HOTAIR expression is upregulated in samples of non-traumatic ONFH, and knockdown of HOTAIR promotes osteogenic differentiation and proliferation. Zhuang et al. [38] have found that MEG3 promotes osteogenic differentiation of MSCs in multiple myeloma. In our study, it was found that NORAD expression was downregulated in SONFH tissues. DEX could inhibit proliferation and induce apoptosis of BMSCs [39, 40]. We investigated the effects of Dex (0, 10^−6^, 10^−7^, and 10^−8^ M) on NORAD expression and proliferation in hBMSCs and found that Dex reduced NORAD expression and inhibited cell proliferation in a dose-dependent manner. In addition, we also found that overexpression of NORAD improved Dex-induced inhibition of proliferation and promotion of apoptosis in hBMSCs, while knockdown of NORAD led to the opposite results. In short, the above findings indicated that NORAD participated in the development of SONFH.

MicroRNAs (miRNAs, usually with 19–25 nucleotides) are small non-coding endogenous RNAs, which can bind to the 3′UTR of target mRNAs and change the stability of mRNA or protein translation, thus regulating various physiological process [41, 42]. In recent decades, there are a lot of researches about the effects of miRNAs on the osteogenesis of MSCs. For instance, Zha et al. [43] have found that miR-34a reduces the inhibitory effects of Dex on mMSCs and osteoblasts. Jia et al. [44] have shown that miR-17-5p facilitates the proliferation and differentiation of HMSC-bm cells by targeting SMAD7. To further analyze the mechanism of NORAD in SONFH, StarBase predicted that NORAD and miR-26a-5p had a binding site. In addition, Luzi et al. [45] have found that miR-26a inhibits terminal differentiation of hADSCs. In this study, the luciferase activity was remarkably reduced; then, cells were co-transfected with miR-26a-5p mimics and NORAD-WT. In addition, miR-26a-5p expression in SONFH was upregulated, and NORAD and miR-26a-5p expression represented a negative correlation. Moreover, NORAD promoted cell proliferation and inhibited cell apoptosis, while these effects were weakened by overexpression of miR-26a-5p. Taken together, these results indicated NORAD improved Dex-induced inhibition of proliferation and promotion of apoptosis by regulation of miR-26a-5p in hBMSCs.

Osteoprotegerin (OPG) is a member of tumor necrosis factor receptor family, which has the function of inhibiting osteoclast differentiation and increasing bone density [46]. OPG can directly bind to RANKL to inhibit the combination between RANKL and RANK, and thus inhibit the activation of osteoclast [47]. OPG/RANK/ RANKL together constitute a key signaling pathway in osteoclast differentiation and bone resorption [48]. In the present study, it was found that Dex reduced the mRNA expression of OPG and increased the mRNA expression of RANK and RANKL in a dose-dependent manner. In addition, overexpression of NORAD improved DEX-induced inhibition of OPG expression and promotion of RANK and RANKL expression, while these effects were weakened by overexpression of miR-26a-5p. RUNX2, as a lineage-specific transcription factor, participates in tumor metastasis and osteogenesis [49–51]. Zhou et al. [52] have demonstrated that overexpression of NORAD increases the mRNA and protein expression of RUNX2, while knockdown of NORAD inhibits the RUNX2 expression in breast cancer cells. In our study, overexpression of NORAD significantly increased the protein expression of RUNX2, but knockdown of NORAD decreased the protein expression of RUNX2. Moreover, transfection of miR-26a-5p mimics remarkably reversed the increase of RUNX2 expression induced by overexpression of NORAD. All in all, the above results suggested that NORAD improved Dex-induced inhibition of differentiation by regulation of miR-26a-5p in hBMSCs.

## Conclusions

NORAD expression was downregulated in SONFH tissues, while miR-26a-5p expression was upregulated. Moreover, NORAD improved Dex-induced inhibition of proliferation and differentiation, and promotion of apoptosis by regulation of miR-26a-5p in hBMSCs. This study may provide a new insight for the treatment and diagnosis of SONFH.

## Data Availability

The datasets used and analyzed during the current study are available from the corresponding author on reasonable request.
